# YOU DON’T KNOW WHAT YOU DON’T KNOW: A SURVEY OF CAREER MENTORING NEEDS IN CLINICAL GEROPSYCHOLOGY

**DOI:** 10.1093/geroni/igz038.3134

**Published:** 2019-11-08

**Authors:** Rachael Spalding, Brenna N Renn, Rebecca S Allen, Jennifer Birdsall, Barry Edelstein

**Affiliations:** 1 West Virginia University, Morgantown, West Virginia, United States; 2 University of Washington School of Medicine, Seattle, Washington, United States; 3 The University of Alabama, Tuscaloosa, Alabama, United States; 4 N/A, N/A, United States

## Abstract

Mentorship carries the potential to improve the placement, persistence, and success of those working in geriatric mental health. We sought to explore the career preferences and perceived barriers to obtaining desired jobs in the field of clinical geropsychology using an online survey of trainees and etablished professional geropsychologists. This cross-sectional observational cohort study recruited 96 respondents .Both trainees (n = 42) and professional geropsychologists (n = 54) completed an online survey. Trainees endorsed a variety of characteristics to describe their ideal jobs in geropsychology; particularly interdisciplinary teamwork and jobs in medical settings, palliative/hospice care facilities, long-term care, and geriatric outpatient care. The most commonly endorsed perceived barrier to trainees’ ideal jobs was the location of the position. Responses from professional geropsychologists elucidated factors that influenced their first job and resources that were helpful, or would have been helpful, during their job search process. The majority of respondents described the role of mentoring in career development, specifically with skill development, decision-making assistance, and personal support. Mentorship is instrumental throughout one’s training and career and offers instrumental and emotional support in job-searching and defining one’s career. Results should be considered with respect to training and retention of clinical geropsychologists given the workforce shortage, particularly in academia.

